# Supplementary material for: Genome-wide survey of single-nucleotide polymorphisms reveals fine-scale population structure and signs of selection in the threatened Caribbean elkhorn coral, Acropora palmata
Source: PeerJ. 2017 Nov 21;5:e4077. doi: 10.7717/peerj.4077 (PMC5701561; doi:10.7717/peerj.4077)
Supplement: Table S1 — There were 12 pools with 8 unique barcodes in each. The Database ID is a unique identifier for each coral specimen. Given is also the total number of ramets for each genet in the Baumslab database. The indices are short DNA sequences that uniquely identify products in the final libraries. [file peerj-05-4077-s009.docx]

| **Pool** | **Barcode** | **Database ID** | **Ramet Count** | **Genet ID** | **PCR2 Indices** |
| --- | --- | --- | --- | --- | --- |
| B1 | AACCA | 9345 | 1 | P2354 | PCR2_Idx_1_ATCACG |
| B1 | AGCTA | 9342 | 1 | P2352 | PCR2_Idx_1_ATCACG |
| B1 | CAACC | 9335 | 1 | P2347 | PCR2_Idx_1_ATCACG |
| B1 | GCATG | 9343 | 1 | P2353 | PCR2_Idx_1_ATCACG |
| B1 | GGTTG | 9340 | 1 | P2350 | PCR2_Idx_1_ATCACG |
| B1 | TGCAT | 9334 | 1 | P2346 | PCR2_Idx_1_ATCACG |
| B2 | AACCA | 9373 | 1 | P2368 | PCR2_Idx_2_CGATGT |
| B2 | AGCTA | 9366 | 1 | P2366 | PCR2_Idx_2_CGATGT |
| B2 | CAACC | 9360 | 1 | P2361 | PCR2_Idx_2_CGATGT |
| B2 | GCATG | 9368 | 1 | P2367 | PCR2_Idx_2_CGATGT |
| B2 | GGTTG | 9363 | 1 | P2363 | PCR2_Idx_2_CGATGT |
| B2 | TGCAT | 9358 | 1 | P2360 | PCR2_Idx_2_CGATGT |
| B3 | AACCA | 1588 | 5 | P1080 | PCR2_Idx_3_TTAGGC |
| B3 | AGCTA | 9328 | 1 | P2345 | PCR2_Idx_3_TTAGGC |
| B3 | CAACC | 9351 | 1 | P2464 | PCR2_Idx_3_TTAGGC |
| B3 | GCATG | 1584 | 9 | P1079 | PCR2_Idx_3_TTAGGC |
| B3 | GGTTG | 9356 | 1 | P2358 | PCR2_Idx_3_TTAGGC |
| B3 | TGCAT | 9350 | 2 | P2355 | PCR2_Idx_3_TTAGGC |
| F1 | AACCA | 6895 | 28 | P1003 | PCR2_Idx_1_ATCACG |
| F1 | AGCTA | 2735 | 1 | P2566 | PCR2_Idx_1_ATCACG |
| F1 | CAACC | 1029 | 25 | P1001 | PCR2_Idx_1_ATCACG |
| F1 | GCATG | 6891 | 4 | P1006 | PCR2_Idx_1_ATCACG |
| F1 | GGTTG | 1157 | 5 | P1021 | PCR2_Idx_1_ATCACG |
| F1 | TGCAT | 1005 | 26 | P1000 | PCR2_Idx_1_ATCACG |
| F2 | AACCA | 5648 | 2 | P2131 | PCR2_Idx_2_CGATGT |
| F2 | AGCTA | 5633 | 1 | P2129 | PCR2_Idx_2_CGATGT |
| F2 | CAACC | 2362 | 1 | P2540 | PCR2_Idx_2_CGATGT |
| F2 | GCATG | 5636 | 1 | P2130 | PCR2_Idx_2_CGATGT |
| F2 | GGTTG | 2699 | 24 | P2564 | PCR2_Idx_2_CGATGT |
| F2 | TGCAT | 2360 | 20 | P2538 | PCR2_Idx_2_CGATGT |
| F3 | AACCA | 6001 | 7 | P1033 | PCR2_Idx_3_TTAGGC |
| F3 | AGCTA | 5588 | 16 | P2123 | PCR2_Idx_3_TTAGGC |
| F3 | CAACC | 2624 | 6 | P1029 | PCR2_Idx_3_TTAGGC |
| F3 | GCATG | 5602 | 28 | P2126 | PCR2_Idx_3_TTAGGC |
| F3 | GGTTG | 2655 | 31 | P1032 | PCR2_Idx_3_TTAGGC |
| F3 | TGCAT | 2614 | 55 | P1028 | PCR2_Idx_3_TTAGGC |
| P1 | AACCA | 6114 | 2 | P2341 | PCR2_Idx_1_ATCACG |
| P1 | AGCTA | 6109 | 1 | P2463 | PCR2_Idx_1_ATCACG |
| P1 | CAACC | 6094 | 1 | P2461 | PCR2_Idx_1_ATCACG |
| P1 | GCATG | 6112 | 5 | P2339 | PCR2_Idx_1_ATCACG |
| P1 | GGTTG | 6101 | 5 | P2334 | PCR2_Idx_1_ATCACG |
| P1 | TGCAT | 6088 | 4 | P2326 | PCR2_Idx_1_ATCACG |
| P2 | AACCA | 6065 | 1 | P2314 | PCR2_Idx_2_CGATGT |
| P2 | AGCTA | 5193 | 1 | P1921 | PCR2_Idx_2_CGATGT |
| P2 | CAACC | 5157 | 1 | P1887 | PCR2_Idx_2_CGATGT |
| P2 | GCATG | 6064 | 3 | P2313 | PCR2_Idx_2_CGATGT |
| P2 | GGTTG | 5191 | 1 | P1919 | PCR2_Idx_2_CGATGT |
| P2 | TGCAT | 5152 | 1 | P1882 | PCR2_Idx_2_CGATGT |
| P3 | AACCA | 5149 | 1 | P1881 | PCR2_Idx_3_TTAGGC |
| P3 | AGCTA | 5140 | 7 | P1878 | PCR2_Idx_3_TTAGGC |
| P3 | CAACC | 5117 | 1 | P1863 | PCR2_Idx_3_TTAGGC |
| P3 | GCATG | 5148 | 1 | P1880 | PCR2_Idx_3_TTAGGC |
| P3 | GGTTG | 5124 | 4 | P1869 | PCR2_Idx_3_TTAGGC |
| P3 | TGCAT | 5109 | 10 | P1857 | PCR2_Idx_3_TTAGGC |
| U1 | AACCA | 4064 | 1 | P1421 | PCR2_Idx_1_ATCACG |
| U1 | AGCTA | 4060 | 2 | P1414 | PCR2_Idx_1_ATCACG |
| U1 | CAACC | 4053 | 2 | P1415 | PCR2_Idx_1_ATCACG |
| U1 | GCATG | 4062 | 1 | P1419 | PCR2_Idx_1_ATCACG |
| U1 | GGTTG | 4056 | 3 | P1417 | PCR2_Idx_1_ATCACG |
| U1 | TGCAT | 4051 | 4 | P1413 | PCR2_Idx_1_ATCACG |
| U2 | AACCA | 4200 | 1 | P2485 | PCR2_Idx_2_CGATGT |
| U2 | AGCTA | 4176 | 6 | P1406 | PCR2_Idx_2_CGATGT |
| U2 | CAACC | 4023 | 5 | P1403 | PCR2_Idx_2_CGATGT |
| U2 | GCATG | 4177 | 1 | P2483 | PCR2_Idx_2_CGATGT |
| U2 | GGTTG | 4044 | 1 | P1410 | PCR2_Idx_2_CGATGT |
| U2 | TGCAT | 4020 | 6 | P1402 | PCR2_Idx_2_CGATGT |
| U3 | AACCA | 1890 | 4 | P2506 | PCR2_Idx_3_TTAGGC |
| U3 | AGCTA | 1878 | 1 | P2512 | PCR2_Idx_3_TTAGGC |
| U3 | CAACC | 1866 | 3 | P2507 | PCR2_Idx_3_TTAGGC |
| U3 | GCATG | 1886 | 7 | P2504 | PCR2_Idx_3_TTAGGC |
| U3 | GGTTG | 1872 | 2 | P2510 | PCR2_Idx_3_TTAGGC |
| U3 | TGCAT | 1862 | 3 | P2505 | PCR2_Idx_3_TTAGGC |
