# Supplementary material for: Genome-wide survey of single-nucleotide polymorphisms reveals fine-scale population structure and signs of selection in the threatened Caribbean elkhorn coral, Acropora palmata
Source: PeerJ. 2017 Nov 21;5:e4077. doi: 10.7717/peerj.4077 (PMC5701561; doi:10.7717/peerj.4077)
Supplement: Table S2 [file peerj-05-4077-s010.docx]

#example Process radtag code for one sample

meghann@meghann-OptiPlex-9020:~$ process_radtags -1 sample1.1.fastq.gz -2 sample1.2.fastq.gz -o /output_folder -b /barcodes.txt -e nlaIII -r -c -q -i gzfastq --adapter_1 AATGATACGGCGACCACCGAGATCTACACTCTTTCCCTACACGACGCTCTTCCGATCT --adapter_mm 2

sudo mysql -e "CREATE DATABASE R1_radtags" -p

sudo mysql R1_radtags < /usr/local/share/stacks/sql/stacks.sql -p

sudo mysql -p

SHOW DATABASES;

USE R1_radtags;

SHOW TABLES;

quit

# Example on how to run STACKs ref_map

meghann@meghann-OptiPlex-9020:~$ ref_map.pl -s /media/BaumsServer/PalmataSNPs/stacks/Bowtie2/Read1/Bowtie2_on_B1_AACCA.1_aligned_reads.bam -s /media/BaumsServer/PalmataSNPs/stacks/Bowtie2/Read1/Bowtie2_on_B1_CAACC.1_aligned_reads.bam -s /media/BaumsServer/PalmataSNPs/stacks/Bowtie2/Read1/Bowtie2_on_B1_AGCTA.1_aligned_reads.bam -s /media/BaumsServer/PalmataSNPs/stacks/Bowtie2/Read1/Bowtie2_on_B1_GCATG.1_aligned_reads.bam -s /media/BaumsServer/PalmataSNPs/stacks/Bowtie2/Read1/Bowtie2_on_B1_GGTTG.1_aligned_reads.bam -s /media/BaumsServer/PalmataSNPs/stacks/Bowtie2/Read1/Bowtie2_on_B1_TGCAT.1_aligned_reads.bam -s /media/BaumsServer/PalmataSNPs/stacks/Bowtie2/Read1/Bowtie2_on_B2_AACCA.1_aligned_reads.bam -s /media/BaumsServer/PalmataSNPs/stacks/Bowtie2/Read1/Bowtie2_on_B2_CAACC.1_aligned_reads.bam -s /media/BaumsServer/PalmataSNPs/stacks/Bowtie2/Read1/Bowtie2_on_B2_AGCTA.1_aligned_reads.bam -s /media/BaumsServer/PalmataSNPs/stacks/Bowtie2/Read1/Bowtie2_on_B2_GCATG.1_aligned_reads.bam -s /media/BaumsServer/PalmataSNPs/stacks/Bowtie2/Read1/Bowtie2_on_B2_GGTTG.1_aligned_reads.bam -s /media/BaumsServer/PalmataSNPs/stacks/Bowtie2/Read1/Bowtie2_on_B2_TGCAT.1_aligned_reads.bam -s /media/BaumsServer/PalmataSNPs/stacks/Bowtie2/Read1/Bowtie2_on_B3_AACCA.1_aligned_reads.bam -s /media/BaumsServer/PalmataSNPs/stacks/Bowtie2/Read1/Bowtie2_on_B3_CAACC.1_aligned_reads.bam -s /media/BaumsServer/PalmataSNPs/stacks/Bowtie2/Read1/Bowtie2_on_B3_AGCTA.1_aligned_reads.bam -s /media/BaumsServer/PalmataSNPs/stacks/Bowtie2/Read1/Bowtie2_on_B3_GCATG.1_aligned_reads.bam -s /media/BaumsServer/PalmataSNPs/stacks/Bowtie2/Read1/Bowtie2_on_B3_GGTTG.1_aligned_reads.bam -s /media/BaumsServer/PalmataSNPs/stacks/Bowtie2/Read1/Bowtie2_on_B3_TGCAT.1_aligned_reads.bam -s /media/BaumsServer/PalmataSNPs/stacks/Bowtie2/Read1/Bowtie2_on_F1_AACCA.1_aligned_reads.bam -s /media/BaumsServer/PalmataSNPs/stacks/Bowtie2/Read1/Bowtie2_on_F1_CAACC.1_aligned_reads.bam -s /media/BaumsServer/PalmataSNPs/stacks/Bowtie2/Read1/Bowtie2_on_F1_AGCTA.1_aligned_reads.bam -s /media/BaumsServer/PalmataSNPs/stacks/Bowtie2/Read1/Bowtie2_on_F1_GCATG.1_aligned_reads.bam -s /media/BaumsServer/PalmataSNPs/stacks/Bowtie2/Read1/Bowtie2_on_F1_GGTTG.1_aligned_reads.bam -s /media/BaumsServer/PalmataSNPs/stacks/Bowtie2/Read1/Bowtie2_on_F1_TGCAT.1_aligned_reads.bam -s /media/BaumsServer/PalmataSNPs/stacks/Bowtie2/Read1/Bowtie2_on_F2_AACCA.1_aligned_reads.bam -s /media/BaumsServer/PalmataSNPs/stacks/Bowtie2/Read1/Bowtie2_on_F2_CAACC.1_aligned_reads.bam -s /media/BaumsServer/PalmataSNPs/stacks/Bowtie2/Read1/Bowtie2_on_F2_AGCTA.1_aligned_reads.bam -s /media/BaumsServer/PalmataSNPs/stacks/Bowtie2/Read1/Bowtie2_on_F2_GCATG.1_aligned_reads.bam -s /media/BaumsServer/PalmataSNPs/stacks/Bowtie2/Read1/Bowtie2_on_F2_GGTTG.1_aligned_reads.bam -s /media/BaumsServer/PalmataSNPs/stacks/Bowtie2/Read1/Bowtie2_on_F2_TGCAT.1_aligned_reads.bam -s /media/BaumsServer/PalmataSNPs/stacks/Bowtie2/Read1/Bowtie2_on_F3_AACCA.1_aligned_reads.bam -s /media/BaumsServer/PalmataSNPs/stacks/Bowtie2/Read1/Bowtie2_on_F3_CAACC.1_aligned_reads.bam -s /media/BaumsServer/PalmataSNPs/stacks/Bowtie2/Read1/Bowtie2_on_F3_AGCTA.1_aligned_reads.bam -s /media/BaumsServer/PalmataSNPs/stacks/Bowtie2/Read1/Bowtie2_on_F3_GCATG.1_aligned_reads.bam -s /media/BaumsServer/PalmataSNPs/stacks/Bowtie2/Read1/Bowtie2_on_F3_GGTTG.1_aligned_reads.bam -s /media/BaumsServer/PalmataSNPs/stacks/Bowtie2/Read1/Bowtie2_on_F3_TGCAT.1_aligned_reads.bam -s /media/BaumsServer/PalmataSNPs/stacks/Bowtie2/Read1/Bowtie2_on_P1_AACCA.1_aligned_reads.bam -s /media/BaumsServer/PalmataSNPs/stacks/Bowtie2/Read1/Bowtie2_on_P1_CAACC.1_aligned_reads.bam -s /media/BaumsServer/PalmataSNPs/stacks/Bowtie2/Read1/Bowtie2_on_P1_AGCTA.1_aligned_reads.bam -s /media/BaumsServer/PalmataSNPs/stacks/Bowtie2/Read1/Bowtie2_on_P1_GCATG.1_aligned_reads.bam -s /media/BaumsServer/PalmataSNPs/stacks/Bowtie2/Read1/Bowtie2_on_P1_GGTTG.1_aligned_reads.bam -s /media/BaumsServer/PalmataSNPs/stacks/Bowtie2/Read1/Bowtie2_on_P1_TGCAT.1_aligned_reads.bam -s /media/BaumsServer/PalmataSNPs/stacks/Bowtie2/Read1/Bowtie2_on_P2_AACCA.1_aligned_reads.bam -s /media/BaumsServer/PalmataSNPs/stacks/Bowtie2/Read1/Bowtie2_on_P2_CAACC.1_aligned_reads.bam -s /media/BaumsServer/PalmataSNPs/stacks/Bowtie2/Read1/Bowtie2_on_P2_AGCTA.1_aligned_reads.bam -s /media/BaumsServer/PalmataSNPs/stacks/Bowtie2/Read1/Bowtie2_on_P2_GCATG.1_aligned_reads.bam -s /media/BaumsServer/PalmataSNPs/stacks/Bowtie2/Read1/Bowtie2_on_P2_GGTTG.1_aligned_reads.bam -s /media/BaumsServer/PalmataSNPs/stacks/Bowtie2/Read1/Bowtie2_on_P2_TGCAT.1_aligned_reads.bam -s /media/BaumsServer/PalmataSNPs/stacks/Bowtie2/Read1/Bowtie2_on_P3_AACCA.1_aligned_reads.bam -s /media/BaumsServer/PalmataSNPs/stacks/Bowtie2/Read1/Bowtie2_on_P3_CAACC.1_aligned_reads.bam -s /media/BaumsServer/PalmataSNPs/stacks/Bowtie2/Read1/Bowtie2_on_P3_AGCTA.1_aligned_reads.bam -s /media/BaumsServer/PalmataSNPs/stacks/Bowtie2/Read1/Bowtie2_on_P3_GCATG.1_aligned_reads.bam -s /media/BaumsServer/PalmataSNPs/stacks/Bowtie2/Read1/Bowtie2_on_P3_GGTTG.1_aligned_reads.bam -s /media/BaumsServer/PalmataSNPs/stacks/Bowtie2/Read1/Bowtie2_on_P3_TGCAT.1_aligned_reads.bam -s /media/BaumsServer/PalmataSNPs/stacks/Bowtie2/Read1/Bowtie2_on_U1_AACCA.1_aligned_reads.bam -s /media/BaumsServer/PalmataSNPs/stacks/Bowtie2/Read1/Bowtie2_on_U1_CAACC.1_aligned_reads.bam -s /media/BaumsServer/PalmataSNPs/stacks/Bowtie2/Read1/Bowtie2_on_U1_AGCTA.1_aligned_reads.bam -s /media/BaumsServer/PalmataSNPs/stacks/Bowtie2/Read1/Bowtie2_on_U1_GCATG.1_aligned_reads.bam -s /media/BaumsServer/PalmataSNPs/stacks/Bowtie2/Read1/Bowtie2_on_U1_GGTTG.1_aligned_reads.bam -s /media/BaumsServer/PalmataSNPs/stacks/Bowtie2/Read1/Bowtie2_on_U1_TGCAT.1_aligned_reads.bam -s /media/BaumsServer/PalmataSNPs/stacks/Bowtie2/Read1/Bowtie2_on_U2_AACCA.1_aligned_reads.bam -s /media/BaumsServer/PalmataSNPs/stacks/Bowtie2/Read1/Bowtie2_on_U2_CAACC.1_aligned_reads.bam -s /media/BaumsServer/PalmataSNPs/stacks/Bowtie2/Read1/Bowtie2_on_U2_AGCTA.1_aligned_reads.bam -s /media/BaumsServer/PalmataSNPs/stacks/Bowtie2/Read1/Bowtie2_on_U2_GCATG.1_aligned_reads.bam -s /media/BaumsServer/PalmataSNPs/stacks/Bowtie2/Read1/Bowtie2_on_U2_GGTTG.1_aligned_reads.bam -s /media/BaumsServer/PalmataSNPs/stacks/Bowtie2/Read1/Bowtie2_on_U2_TGCAT.1_aligned_reads.bam -s /media/BaumsServer/PalmataSNPs/stacks/Bowtie2/Read1/Bowtie2_on_U3_AACCA.1_aligned_reads.bam -s /media/BaumsServer/PalmataSNPs/stacks/Bowtie2/Read1/Bowtie2_on_U3_CAACC.1_aligned_reads.bam -s /media/BaumsServer/PalmataSNPs/stacks/Bowtie2/Read1/Bowtie2_on_U3_AGCTA.1_aligned_reads.bam -s /media/BaumsServer/PalmataSNPs/stacks/Bowtie2/Read1/Bowtie2_on_U3_GCATG.1_aligned_reads.bam -s /media/BaumsServer/PalmataSNPs/stacks/Bowtie2/Read1/Bowtie2_on_U3_GGTTG.1_aligned_reads.bam -s /media/BaumsServer/PalmataSNPs/stacks/Bowtie2/Read1/Bowtie2_on_U3_TGCAT.1_aligned_reads.bam -o /media/BaumsServer/PalmataSNPs/stacks -n 4 -m 5 -T 8 -B R1_radtags -O /media/BaumsServer/PalmataSNPs/PopulationFiles/PopulationMap_4.txt -b 1 -D Fl_BAH_PR_USVI

populations -b 1 -P /media/BaumsServer/PalmataSNPs/stacks -M /media/BaumsServer/PalmataSNPs/PopulationFiles/PopulationMap_4.txt -r 0.6 -m 5 -f p_value -p 4 --fasta --genepop --structure --lnl_lim -10.0 -a 0.05 --fstats --write_single_snp
