# Supplementary material for: Genome-wide survey of single-nucleotide polymorphisms reveals fine-scale population structure and signs of selection in the threatened Caribbean elkhorn coral, Acropora palmata
Source: PeerJ. 2017 Nov 21;5:e4077. doi: 10.7717/peerj.4077 (PMC5701561; doi:10.7717/peerj.4077)
Supplement: Table S3 [file peerj-05-4077-s011.docx]

| Region | Latitude | Longitude | Environmental Condition | Grid |
| --- | --- | --- | --- | --- |
| Bahamas | 22.125 | -75.125 | Average Temp | 1/4 degree |
|  | 22.375 | -75.625 | Average Temp | 1/4 degree |
|  | 23.625 | -76.625 | Average Temp | 1/4 degree |
|  | 23.5 | -76.5 | Dissolved Oxygen | 1/4 degree |
|  | -22.5 | -75.5 | Dissolved Oxygen | 1/4 degree |
|  | 22.125 | -75.125 | Salinity | 1 degree |
|  | 22.375 | -75.125 | Salinity | 1 degree |
|  | 23.625 | -76.625 | Salinity | 1 degree |
|  | 23.5 | -76.5 | phosphates | 1 degree |
|  | 22.5 | -74.5 | phosphates | 1 degree |
| Florida | 25.125 | -80.125 | Average Temp | 1/4 degree |
|  | 25.5 | -79.5 | Dissolved Oxygen | 1/4 degree |
|  | 25.125 | -80.125 | Salinity | 1 degree |
|  | 25.5 | -79.5 | phosphates | 1 degree |
| Puerto Rico | 18.125 | -67.375 | Average Temp | 1/4 degree |
|  | 18.375 | -67.375 | Average Temp | 1/4 degree |
|  | 18.375 | -65.375 | Average Temp | 1/4 degree |
|  | 17.625 | -67.125 | Average Temp | 1/4 degree |
|  | 18.5 | -67.5 | Dissolved Oxygen | 1/4 degree |
|  | 17.5 | -67.5 | Dissolved Oxygen | 1/4 degree |
|  | 18.125 | -67.375 | Salinity | 1 degree |
|  | 18.375 | -67.375 | Salinity | 1 degree |
|  | 18.375 | -65.375 | Salinity | 1 degree |
|  | 17.625 | -67.125 | Salinity | 1 degree |
|  | 17.5 | -67.5 | phosphates | 1 degree |
|  | 18.5 | -67.5 | phosphates | 1 degree |
| U.S. Virgin Islands | 18.375 | -64.875 | Average Temp | 1/4 degree |
|  | 17.875 | -64.625 | Average Temp | 1/4 degree |
|  | 17.5 | -64.5 | Dissolved Oxygen | 1/4 degree |
|  | 18.5 | -64.5 | Dissolved Oxygen | 1/4 degree |
|  | 18.375 | -64.875 | Salinity | 1 degree |
|  | 17.875 | -64.625 | Salinity | 1 degree |
|  | 17.5 | -64.5 | phosphates | 1 degree |
|  | 18.5 | -64.5 | phosphates | 1 degree |
