# Supplementary material for: Genome-wide survey of single-nucleotide polymorphisms reveals fine-scale population structure and signs of selection in the threatened Caribbean elkhorn coral, Acropora palmata
Source: PeerJ. 2017 Nov 21;5:e4077. doi: 10.7717/peerj.4077 (PMC5701561; doi:10.7717/peerj.4077)
Supplement: Table S4 — Stacks locus_bp is the Stacks program locus ID with the SNP location basepair after the underscore. Read category indicates whether the outlier SNP was found in read 1 or read 2 or the paired-end sequencing run. A. dig scaffold identifies the A. digitifera scaffold where the A. palmata Stacks locus aligned to, followed by the basepair location in the next column. S start, sequence start; S end, sequence end; Lositan P, Lositan P-values (Simulated FST < sample FST). Bayescan q, Bayescan q-values; Ns, not significant. [file peerj-05-4077-s012.docx]

| **Stacks locus_bp** | **R** | **Sequence** | **A.dig Scaffold** | **A.dig bp** | **Annotation** | **Blast Hit** | **% ID** | **s. start** | **s. end** | **e-value** | **bit** | **Lositan P** | **Bayescan q** |
| --- | --- | --- | --- | --- | --- | --- | --- | --- | --- | --- | --- | --- | --- |
| 13890_125 | 1 | CATGACCTCGCTATCGCTGTTCGTTTGCTCTTACGAAGCACGATGTCTATGTGAAGGCGGTCATTAGCATATATCGAAGAGGGCAGGAAAAACCGATCGAGAGATACAAAGCACGCATTTGAGTCTCTGAAGACAGTACAANNNN | gi\|342259630\|dbj\|BACK01037465.1\| | 3159 |  |  |  |  |  |  |  | 1.000 | ns |
| 14348_52 | 1 | CATGAACACATTCAAGATACCTGAATAATGACGGCAGCACTTCGTTGTACGACAAAATGCCTTCTCTGCAAAACTCCCCTGATCCATCGTTGCAGAATACAGATCTTATTAACCTGTTCGGAGTGAAGACGCGACTGCAGCAGTG | gi\|342260087\|dbj\|BACK01037008.1\| | 216 | PREDICTED: unconventional myosin-IXb isoform X7[Mustela putorius furo] | gi\|859823983\|ref\|XP_012916007.1\| | 60 | 941 | 980 | 1.72E-06 | 53.9 | 1.000 | ns |
| 16962_117 | 1 | CATGCTTGACAGTCTCGATGAGTTCGTCTCTCCGAAATGCAAACATTACGTTCTTCCAACTTTGTGCAACTATGTCTTCCCGCCGTGTGATACCTCGCATTCTGAACCAAAACCGAGGAAGTTTTGCCGAGGGGACTGTTTTTTG | gi\|342262202\|dbj\|BACK01034893.1\| | 11174 | PREDICTED: tyrosine-protein kinase transmembrane receptor ROR1-like [Gavia stellata] | gi\|698448314\|ref\|XP_009818383.1\| | 50 | 111 | 148 | 1.85E-06 | 51.6 | 0.995 | ns |
| 18762_37 | 1 | CATGACTGTAATAGGACAAAGAGTATGAAACACTTGTTCCTTTTTGTAGCGTATCAAACTATGAGTAAGTGAAGACACAGATAAGGAGTATGCGTCAGGGTTCCACTATACTATTTCAACAAACGAGAGTCATCTTCTTTTAATA | gi\|342263849\|dbj\|BACK01033246.1\| | 5520 |  |  |  |  |  |  |  | 0.998 | ns |
| 28910_137 | 1 | CATGTCCAGCGTGTGAATCATCGCGTTGCGTTGTACAAACGCGCACATCAAGCTATCGTGGAGAAACAAAAGCCATATGAAGAAGGCCAGGGATGGTTGAGAAATGATAGCAACAATATGCTTGAGCCGATTTGGTCCTGTGGAG | gi\|342272621\|dbj\|BACK01024474.1\| | 4640 | predicted protein [Nematostella vectensis] | gi\|156400068\|ref\|XP_001638822.1\|;gi\|156225946\|gb\|EDO46759.1\| | 64.583 | 38 | 84 | 4.43E-14 | 75.9 | 1.000 | ns |
| 34767_135 | 1 | CATGAAACATTGTGAACGAAAGTACATTTCTCGGTTACTTGTCCTTGGACAACCTGACCATCGCTCCAAAATGCCAAAAAAGCATAGTGCAGAAATAGACCATTTCTCTTGTCTTTTCTTCAAATCGAGTGCTTAGATGTNNNNN | gi\|342276797\|dbj\|BACK01020298.1\| | 6155 |  |  |  |  |  |  |  | 1.000 | ns |
| 80994_17 | 1 | CATGGAAATCGCTTTGGAAAAGAGGCTATTCCGCTCCCAAAGCAGGTTTTGATATTCGTGTGGTTCATCTCAAACCTGGAAGCAACGAGATCTGTTTCTGACAAAAAAGAAGGGCTATATCAGGCGCCAAAAGCCCACACGCTCC | gi\|342271542\|dbj\|BACK01025553.1\| | 5143 |  |  |  |  |  |  |  | 0.999 | 0.027 |
| 21009_114 | 2 | AATTGGCAAAGTGAAAAAAGGCAATGGCTTCTACACAAAGAAGCTCACTGAAACCTTGAAACAGGATGAGTATGGTGACTGGTATATGGGTTAGCACAAAATATCAGTTTCTGAAGCTGCCAAAGCTCAGGCAATCAAGAGGAGAGATTC | gi\|342267929\|dbj\|BACK01029166.1\| | 5088 |  |  |  |  |  |  |  | 0.993 | ns |
| 37218_109 | 2 | AATTTAGTAGTCTATTAGCCTTTGGTAATGCTCTTAAGTGGCTGTAACAAACAGGTTACCATAAAATGCTGGTCGTGATTTCCGTCTGTGGAAAGAAAAAAACTCCTCTCTTTCTTCTTGTTGCCTTTTAGATCGCAACTCGCTCATCNN | gi\|342281972\|dbj\|BACK01015123.1\| | 2510 |  |  |  |  |  |  |  | 1.000 | ns |
| 47222_56 | 2 | AATTTGACGAAAAATCAATCACAACTAACCAAGGAAAGTTTGGTATCAAACGACTTCCTAAGGGTAAAACTGATTTACACCGAAAAAAAACTACGAAAACGACGTTTGCAGCGATAGCCCTTCGTCAGAGCGAACTAAAAAACCTGAGTA | gi\|342289964\|dbj\|BACK01007131.1\| | 1947 |  |  |  |  |  |  |  | 1.000 | ns |
| 50064_146 | 2 | AATTTTTAGCAAAATGTTGCAAAAAGTATGGCATTTGGTGAAAAACCACCAAAACTTGTCGATCTCCTCGAAATAAGCAACTTTTTGTGCAAAGTACCGTGCACTAGGGTTTTGACCCAATTTTTAGAAAAATGTTGCAAAAAGTATGGC | gi\|342292085\|dbj\|BACK01005010.1\| | 8821 |  |  |  |  |  |  |  | 0.997 | ns |
| 89179_143 | 2 | AATTTGCCCTCTACTTGCGTAATGGTCTGAGTTTGAGCACTGCTTCCAGAAAACGCGCTATGAACGGCTTTTCAAAGAAGGTTACTTAACTTATACCGAAACCTCTTTTTAGGCACTGAAAACACCCAGAAAAGTGCTACAACCGACATT | gi\|342293160\|dbj\|BACK01003935.1\| | 25161 |  |  |  |  |  |  |  | 1.000 | ns |
| 50062_122 | 2 | AATTTTTAGCAAAATGTTGCAAAAAGTATGGCATTTGGTGAAAAACCACCAAAACTTGTCGATCTCCTCGAAATAAGCAACTTTTTGTGCAAAGTACCGTGCACTAGGGTTTTGACCCAATTTTTAGAAAAATGTTGCAAAAAGTATGGC | gi\|342292085\|dbj\|BACK01005010.1\| | 8968 |  |  |  |  |  |  |  | NA | 0.006 |
