# Supplementary material for: Genome-wide survey of single-nucleotide polymorphisms reveals fine-scale population structure and signs of selection in the threatened Caribbean elkhorn coral, Acropora palmata
Source: PeerJ. 2017 Nov 21;5:e4077. doi: 10.7717/peerj.4077 (PMC5701561; doi:10.7717/peerj.4077)
Supplement: Table S5 — The Database ID is a unique identifier for each coral specimen. Given is also is the geographic region and reef the samples were collected from, microsatellite allele call 1 and 2 for loci 166, 181, 182, 192, and 207 (in basebairs), and the coral genet ID. The last four columns show how the radseq samples were pooled and gives the inline barcode, the Illumina index, the Illumina sequence lane and chip. [file peerj-05-4077-s013.docx]

| **Region** | **Reef** | **Database ID** | **166_1** | **166_2** | **181_1** | **181_2** | **182_1** | **182_2** | **192_1** | **192_2** | **207_1** | **207_2** | **Genet ID** | **Pool** | **Barcode** | **Ilumina Index** | **Lane** | **Illumina Chip** |
| --- | --- | --- | --- | --- | --- | --- | --- | --- | --- | --- | --- | --- | --- | --- | --- | --- | --- | --- |
| Bahamas | Adelaine Cay | 9345 | 161 | 161 | 153 | 156 | 143 | 188 | 172 | 181 | 179 | 200 | P2354 | B1 | AACCA | ATCACG | 1 | 2 |
| Bahamas | Adelaine Cay | 9342 | 152 | 170 | 156 | 156 | 167 | 170 | 157 | 160 | 182 | 188 | P2352 | B1 | AGCTA | ATCACG | 1 | 2 |
| Bahamas | Adelaine Cay | 9335 | 155 | 158 | 156 | 159 | 173 | 194 | 160 | 169 | 173 | 176 | P2347 | B1 | CAACC | ATCACG | 1 | 2 |
| Bahamas | Adelaine Cay | 9339 | 164 | 167 | 156 | 156 | 167 | 167 | 169 | 178 | 179 | 191 | P2349 | B1 | CAACC | ATCACG | 1 | 2 |
| Bahamas | Adelaine Cay | 9343 | 161 | 176 | 156 | 162 | 143 | 173 | 175 | 178 | 176 | 182 | P2353 | B1 | GCATG | ATCACG | 1 | 2 |
| Bahamas | Adelaine Cay | 9340 | 161 | 173 | 156 | 156 | 170 | 185 | 151 | 163 | 176 | 179 | P2350 | B1 | GGTTG | ATCACG | 1 | 2 |
| Bahamas | Adelaine Cay | 9341 | 170 | 182 | 156 | 162 | 149 | 191 | 154 | 169 | 176 | 185 | P2351 | B1 | TCGAT | ATCACG | 1 | 2 |
| Bahamas | Adelaine Cay | 9334 | 143 | 158 | 156 | 159 | 143 | 185 | 154 | 172 | 170 | 176 | P2346 | B1 | TGCAT | ATCACG | 1 | 2 |
| Bahamas | Nairn Cay | 9373 | 161 | 167 | 156 | 162 | 143 | 143 | 169 | 172 | 182 | 197 | P2368 | B2 | AACCA | CGATGT | 1 | 2 |
| Bahamas | Nairn Cay | 9366 | 164 | 176 | 156 | 156 | 143 | 152 | 157 | 157 | 173 | 176 | P2366 | B2 | AGCTA | CGATGT | 1 | 2 |
| Bahamas | Nairn Cay | 9360 | 155 | 164 | 156 | 162 | 170 | 179 | 169 | 169 | 173 | 173 | P2361 | B2 | CAACC | CGATGT | 1 | 2 |
| Bahamas | Nairn Cay | 9362 | 137 | 164 | 156 | 156 | 170 | 191 | 142 | 160 | 179 | 200 | P2362 | B2 | CGATC | CGATGT | 1 | 2 |
| Bahamas | Nairn Cay | 9368 | 146 | 164 | 156 | 156 | 167 | 179 | 172 | 178 | 179 | 185 | P2367 | B2 | GCATG | CGATGT | 1 | 2 |
| Bahamas | Nairn Cay | 9363 | 158 | 161 | 156 | 162 | 143 | 143 | 157 | 163 | 176 | 179 | P2363 | B2 | GGTTG | CGATGT | 1 | 2 |
| Bahamas | Nairn Cay | 9365 | 164 | 173 | 156 | 162 | 143 | 164 | 160 | 160 | 173 | 179 | P2365 | B2 | TCGAT | CGATGT | 1 | 2 |
| Bahamas | Nairn Cay | 9358 | 164 | 173 | 156 | 156 | 167 | 170 | 163 | 163 | 179 | 185 | P2360 | B2 | TGCAT | CGATGT | 1 | 2 |
| Bahamas | Elkorn Cay | 9351 | 170 | 176 | 156 | 156 | 143 | 170 | 157 | 166 | 173 | 173 | P2464 | B3 | CAACC | TTAGGC | 1 | 2 |
| Bahamas | Elkorn Cay | 9350 | 164 | 167 | 156 | 156 | 155 | 155 | 151 | 166 | 179 | 185 | P2355 | B3 | TGCAT | TTAGGC | 1 | 2 |
| Bahamas | Johnson Cay | 9355 | 140 | 167 | 156 | 156 | 155 | 182 | 163 | 166 | 185 | 197 | P2357 | B3 | CGATC | TTAGGC | 1 | 2 |
| Bahamas | Johnson Cay | 9356 | 164 | 167 | 156 | 162 | 155 | 188 | 166 | 211 | 170 | 179 | P2358 | B3 | GGTTG | TTAGGC | 1 | 2 |
| Bahamas | Johnson Cay | 9357 | 155 | 167 | 156 | 156 | 155 | 167 | 169 | 178 | 176 | 194 | P2359 | B3 | TCGAT | TTAGGC | 1 | 2 |
| Bahamas | Little Ragged Island | 9328 | 158 | 167 | 159 | 159 | 158 | 170 | 175 | 175 | 167 | 200 | P2345 | B3 | AGCTA | TTAGGC | 1 | 2 |
| Bahamas | Middle Beach | 1588 | 155 | 176 | 156 | 156 | 143 | 167 | 169 | 181 | 179 | 179 | P1080 | B3 | AACCA | TTAGGC | 1 | 2 |
| Bahamas | Middle Beach | 1584 | 155 | 173 | 156 | 156 | 143 | 170 | 172 | 172 | 176 | 179 | P1079 | B3 | GCATG | TTAGGC | 1 | 2 |
| Florida | Horseshoe | 1005 | 164 | 170 | 156 | 156 | 143 | 170 | 160 | 169 | 173 | 173 | P1000 | F1 | TGCAT | ATCACG | 1 | 1 |
| Florida | Little Grecian | 1029 | 170 | 173 | 156 | 156 | 143 | 143 | 163 | 175 | 173 | 176 | P1001 | F1 | CAACC | ATCACG | 1 | 1 |
| Florida | Sand Island | 6895 | 170 | 170 | 156 | 159 | 158 | 191 | 145 | 172 | 173 | 176 | P1003 | F1 | AACCA | ATCACG | 1 | 1 |
| Florida | Sand Island | 2735 | 143 | 167 | 153 | 156 | 143 | 155 | 139 | 172 | 176 | 185 | P2566 | F1 | AGCTA | ATCACG | 1 | 1 |
| Florida | Sand Island | 1082 | 149 | 173 | 156 | 159 | 170 | 173 | 151 | 151 | 173 | 179 | P1002 | F1 | CGATC | ATCACG | 1 | 1 |
| Florida | Sand Island | 1157 | 149 | 170 | 156 | 156 | 143 | 146 | 166 | 166 | 161 | 182 | P1021 | F1 | GGTTG | ATCACG | 1 | 1 |
| Florida | Sand Island | 1168 | 158 | 176 | 159 | 165 | 143 | 152 | 160 | 166 | 161 | 200 | P1023 | F1 | TCGAT | ATCACG | 1 | 1 |
| Florida | Sand Island | 6891 | 164 | 173 | 156 | 156 | 143 | 176 | 163 | 166 | 185 | 188 | P1006 | F1 | GCATG | ATCACG | 1 | 1 |
| Florida | French | 2362 | 167 | 170 | 156 | 156 | 167 | 194 | 151 | 157 | 185 | 197 | P2540 | F2 | CAACC | CGATGT | 1 | 1 |
| Florida | French | 2360 | 170 | 179 | 156 | 162 | 152 | 176 | 163 | 166 | 173 | 176 | P2538 | F2 | TGCAT | CGATGT | 1 | 1 |
| Florida | French | 5648 | 164 | 170 | 156 | 156 | 140 | 158 | 151 | 160 | 176 | 185 | P2131 | F2 | AACCA | CGATGT | 1 | 1 |
| Florida | French | 5633 | 146 | 158 | 156 | 159 | 164 | 167 | 163 | 172 | 173 | 182 | P2129 | F2 | AGCTA | CGATGT | 1 | 1 |
| Florida | French | 2669 | 143 | 167 | 156 | 156 | 176 | 188 | 163 | 175 | 173 | 176 | P2128 | F2 | CGATC | CGATGT | 1 | 1 |
| Florida | French | 5636 | 182 | 182 | 156 | 156 | 146 | 170 | 169 | 169 | 173 | 173 | P2130 | F2 | GCATG | CGATGT | 1 | 1 |
| Florida | French | 2699 | 149 | 167 | 156 | 156 | 140 | 182 | 151 | 166 | 176 | 188 | P2564 | F2 | GGTTG | CGATGT | 1 | 1 |
| Florida | French | 2849 | 176 | 176 | 156 | 156 | 179 | 188 | 166 | 169 | 164 | 185 | P2539 | F2 | TCGAT | CGATGT | 1 | 1 |
| Florida | Elbow | 2624 | 155 | 170 | 156 | 156 | 143 | 173 | 166 | 175 | 182 | 185 | P1029 | F3 | CAACC | TTAGGC | 1 | 1 |
| Florida | Elbow | 2643 | 170 | 185 | 156 | 165 | 158 | 173 | 175 | 205 | 161 | 173 | P1030 | F3 | CGATC | TTAGGC | 1 | 1 |
| Florida | Elbow | 2614 | 158 | 167 | 156 | 159 | 164 | 167 | 160 | 163 | 173 | 173 | P1028 | F3 | TGCAT | TTAGGC | 1 | 1 |
| Florida | Elbow | 6001 | 140 | 161 | 156 | 159 | 152 | 167 | 166 | 169 | 173 | 185 | P1033 | F3 | AACCA | TTAGGC | 1 | 1 |
| Florida | Elbow | 2655 | 158 | 164 | 156 | 177 | 143 | 167 | 157 | 163 | 173 | 191 | P1032 | F3 | GGTTG | TTAGGC | 1 | 1 |
| Florida | Elbow | 5588 | 152 | 170 | 156 | 159 | 143 | 152 | 163 | 172 | 173 | 182 | P2123 | F3 | AGCTA | TTAGGC | 1 | 1 |
| Florida | Elbow | 2658 | 149 | 173 | 156 | 156 | 170 | 188 | 163 | 166 | 179 | 179 | P2122 | F3 | TCGAT | TTAGGC | 1 | 1 |
| Florida | Elbow | 5602 | 167 | 170 | 156 | 159 | 143 | 167 | 163 | 163 | 173 | 176 | P2126 | F3 | GCATG | TTAGGC | 1 | 1 |
| PuertoRico | La Cordillera | 6094 | 119 | 143 | 156 | 156 | 143 | 158 | 163 | 166 | 176 | 176 | P2461 | P1 | CAACC | ATCACG | 2 | 1 |
| PuertoRico | La Cordillera | 6098 | 143 | 155 | 156 | 156 | 143 | 143 | 151 | 178 | 176 | 179 | P2332 | P1 | CGATC | ATCACG | 2 | 1 |
| PuertoRico | La Cordillera | 6088 | 149 | 176 | 153 | 156 | 143 | 143 | 151 | 166 | 176 | 185 | P2326 | P1 | TGCAT | ATCACG | 2 | 1 |
| PuertoRico | La Cordillera | 6109 | 161 | 182 | 156 | 156 | 143 | 170 | 175 | 178 | 176 | 182 | P2463 | P1 | AGCTA | ATCACG | 2 | 1 |
| PuertoRico | La Cordillera | 6101 | 170 | 173 | 156 | 156 | 143 | 143 | 166 | 172 | 164 | 170 | P2334 | P1 | GGTTG | ATCACG | 2 | 1 |
| PuertoRico | La Cordillera | 6107 | 167 | 176 | 156 | 156 | 143 | 167 | 151 | 166 | 164 | 173 | P2336 | P1 | TCGAT | ATCACG | 2 | 1 |
| PuertoRico | La Cordillera | 6114 | 155 | 170 | 156 | 156 | 158 | 191 | 166 | 175 | 182 | 191 | P2341 | P1 | AACCA | ATCACG | 2 | 1 |
| PuertoRico | La Cordillera | 6112 | 143 | 143 | 156 | 156 | 143 | 152 | 157 | 157 | 179 | 179 | P2339 | P1 | GCATG | ATCACG | 2 | 1 |
| PuertoRico | Rincon | 5193 | 143 | 164 | 156 | 159 | 143 | 143 | 169 | 178 | 173 | 179 | P1921 | P2 | AGCTA | CGATGT | 2 | 1 |
| PuertoRico | Rincon | 5157 | 179 | 179 | 156 | 156 | 140 | 158 | 151 | 151 | 173 | 176 | P1887 | P2 | CAACC | CGATGT | 2 | 1 |
| PuertoRico | Rincon | 5172 | 155 | 164 | 156 | 156 | 167 | 173 | 163 | 166 | 182 | 182 | P1901 | P2 | CGATC | CGATGT | 2 | 1 |
| PuertoRico | Rincon | 5191 | 128 | 182 | 153 | 156 | 143 | 143 | 160 | 166 | 173 | 200 | P1919 | P2 | GGTTG | CGATGT | 2 | 1 |
| PuertoRico | Rincon | 5176 | 164 | 167 | 156 | 159 | 143 | 143 | 163 | 178 | 179 | 179 | P1905 | P2 | TCGAT | CGATGT | 2 | 1 |
| PuertoRico | Rincon | 5152 | 143 | 170 | 156 | 156 | 143 | 176 | 154 | 169 | 176 | 188 | P1882 | P2 | TGCAT | CGATGT | 2 | 1 |
| PuertoRico | Tres Palmas | 6065 | 161 | 185 | 156 | 156 | 143 | 155 | 139 | 166 | 179 | 179 | P2314 | P2 | AACCA | CGATGT | 2 | 1 |
| PuertoRico | Tres Palmas | 6064 | 170 | 173 | 156 | 156 | 143 | 167 | 154 | 172 | 164 | 179 | P2313 | P2 | GCATG | CGATGT | 2 | 1 |
| PuertoRico | San Cristobal | 5149 | 170 | 179 | 156 | 159 | 143 | 143 | 175 | 181 | 164 | 179 | P1881 | P3 | AACCA | TTAGGC | 2 | 1 |
| PuertoRico | San Cristobal | 5140 | 161 | 170 | 156 | 156 | 143 | 176 | 151 | 169 | 176 | 176 | P1878 | P3 | AGCTA | TTAGGC | 2 | 1 |
| PuertoRico | San Cristobal | 5117 | 134 | 158 | 156 | 159 | 143 | 155 | 148 | 181 | 176 | 197 | P1863 | P3 | CAACC | TTAGGC | 2 | 1 |
| PuertoRico | San Cristobal | 5121 | 128 | 158 | 156 | 162 | 158 | 182 | 151 | 154 | 173 | 176 | P1867 | P3 | CGATC | TTAGGC | 2 | 1 |
| PuertoRico | San Cristobal | 5148 | 161 | 185 | 156 | 156 | 143 | 176 | 151 | 163 | 167 | 170 | P1880 | P3 | GCATG | TTAGGC | 2 | 1 |
| PuertoRico | San Cristobal | 5124 | 164 | 173 | 156 | 156 | 143 | 179 | 157 | 166 | 173 | 185 | P1869 | P3 | GGTTG | TTAGGC | 2 | 1 |
| PuertoRico | San Cristobal | 5130 | 131 | 143 | 156 | 156 | 155 | 167 | 160 | 166 | 176 | 182 | P1872 | P3 | TCGAT | TTAGGC | 2 | 1 |
| PuertoRico | San Cristobal | 5109 | 152 | 173 | 156 | 159 | 143 | 179 | 154 | 175 | 173 | 182 | P1857 | P3 | TGCAT | TTAGGC | 2 | 1 |
| USVI | Johnsons Reef | 4064 | 143 | 143 | 156 | 156 | 143 | 167 | 151 | 163 | 179 | 188 | P1421 | U1 | AACCA | ATCACG | 2 | 2 |
| USVI | Johnsons Reef | 4060 | 131 | 137 | 156 | 156 | 143 | 143 | 157 | 172 | 176 | 194 | P1414 | U1 | AGCTA | ATCACG | 2 | 2 |
| USVI | Johnsons Reef | 4053 | 146 | 149 | 156 | 156 | 143 | 176 | 157 | 163 | 176 | 179 | P1415 | U1 | CAACC | ATCACG | 2 | 2 |
| USVI | Johnsons Reef | 4054 | 131 | 146 | 156 | 156 | 143 | 182 | 157 | 175 | 179 | 179 | P1416 | U1 | CGATC | ATCACG | 2 | 2 |
| USVI | Johnsons Reef | 4062 | 143 | 143 | 156 | 156 | 158 | 167 | 151 | 157 | 176 | 185 | P1419 | U1 | GCATG | ATCACG | 2 | 2 |
| USVI | Johnsons Reef | 4056 | 131 | 134 | 156 | 159 | 158 | 167 | 163 | 178 | 173 | 173 | P1417 | U1 | GGTTG | ATCACG | 2 | 2 |
| USVI | Johnsons Reef | 4058 | 143 | 149 | 156 | 159 | 164 | 167 | 166 | 175 | 176 | 185 | P1418 | U1 | TCGAT | ATCACG | 2 | 2 |
| USVI | Johnsons Reef | 4051 | 143 | 143 | 156 | 156 | 143 | 158 | 160 | 178 | 176 | 176 | P1413 | U1 | TGCAT | ATCACG | 2 | 2 |
| USVI | Hawksnest Bay | 4023 | 149 | 149 | 156 | 156 | 143 | 191 | 151 | 163 | 176 | 185 | P1403 | U2 | CAACC | CGATGT | 2 | 2 |
| USVI | Hawksnest Bay | 4020 | 137 | 179 | 156 | 156 | 143 | 158 | 166 | 172 | 176 | 179 | P1402 | U2 | TGCAT | CGATGT | 2 | 2 |
| USVI | Hawksnest Bay | 4041 | 143 | 146 | 156 | 156 | 143 | 167 | 151 | 178 | 173 | 185 | P1409 | U2 | CGATC | CGATGT | 2 | 2 |
| USVI | Hawksnest Bay | 4044 | 143 | 152 | 156 | 156 | 143 | 170 | 157 | 172 | 182 | 188 | P1410 | U2 | GGTTG | CGATGT | 2 | 2 |
| USVI | Hawksnest Bay | 4200 | 146 | 149 | 156 | 183 | 143 | 143 | 172 | 178 | 176 | 179 | P2485 | U2 | AACCA | CGATGT | 2 | 2 |
| USVI | Hawksnest Bay | 4176 | 143 | 146 | 156 | 159 | 143 | 155 | 151 | 160 | 185 | 188 | P1406 | U2 | AGCTA | CGATGT | 2 | 2 |
| USVI | Hawksnest Bay | 4177 | 146 | 146 | 156 | 156 | 143 | 158 | 178 | 178 | 176 | 176 | P2483 | U2 | GCATG | CGATGT | 2 | 2 |
| USVI | Hawksnest Bay | 4147 | 143 | 149 | 156 | 156 | 143 | 143 | 154 | 166 | 182 | 182 | P1399 | U2 | TCGAT | CGATGT | 2 | 2 |
| USVI | Tague Bay | 1890 | 143 | 143 | 156 | 156 | 143 | 167 | 163 | 169 | 173 | 176 | P2506 | U3 | AACCA | TTAGGC | 2 | 2 |
| USVI | Tague Bay | 1878 | 143 | 143 | 156 | 156 | 143 | 179 | 163 | 169 | 164 | 179 | P2512 | U3 | AGCTA | TTAGGC | 2 | 2 |
| USVI | Tague Bay | 1866 | 143 | 143 | 156 | 156 | 143 | 155 | 166 | 175 | 176 | 176 | P2507 | U3 | CAACC | TTAGGC | 2 | 2 |
| USVI | Tague Bay | 1869 | 143 | 143 | 156 | 156 | 146 | 167 | 154 | 157 | 167 | 197 | P2508 | U3 | CGATC | TTAGGC | 2 | 2 |
| USVI | Tague Bay | 1886 | 143 | 149 | 156 | 156 | 143 | 182 | 166 | 178 | 173 | 188 | P2504 | U3 | GCATG | TTAGGC | 2 | 2 |
| USVI | Tague Bay | 1872 | 134 | 140 | 156 | 156 | 143 | 167 | 151 | 169 | 176 | 200 | P2510 | U3 | GGTTG | TTAGGC | 2 | 2 |
| USVI | Tague Bay | 1877 | 146 | 146 | 156 | 162 | 143 | 182 | 151 | 169 | 176 | 185 | P2511 | U3 | TCGAT | TTAGGC | 2 | 2 |
| USVI | Tague Bay | 1862 | 137 | 143 | 156 | 156 | 143 | 143 | 151 | 163 | 173 | 176 | P2505 | U3 | TGCAT | TTAGGC | 2 | 2 |
